# A prospective descriptive study of cryptococcal meningitis in HIV uninfected patients in Vietnam - high prevalence of *Cryptococcus neoformans var grubii *in the absence of underlying disease

**DOI:** 10.1186/1471-2334-10-199

**Published:** 2010-07-09

**Authors:** Tran TH Chau, Nguyen H Mai, Nguyen H Phu, Ho D Nghia, Ly V Chuong, Dinh X Sinh, Van A Duong, Pham T Diep, James I Campbell, Stephen Baker, Tran T Hien, David G Lalloo, Jeremy J Farrar, Jeremy N Day

**Affiliations:** 1 Hospital for Tropical Diseases, 190 Ben Ham Tu, Quan 5, Ho Chi Minh City, Vietnam; 2 Oxford University Clinical Research Unit, Hospital for Tropical Diseases, 190 Ben Ham Tu, Quan 5, Ho Chi Minh City, Vietnam; 3 Centre for Tropical Medicine, Nuffield Department of Clinical Medicine, Oxford University, Oxford, OX3 7LJ, UK; 4 Liverpool School of Tropical Medicine, Liverpool, L3 5QA, UK

## Abstract

**Background:**

Most cases of cryptococcal meningitis occur in patients with HIV infection: the course and outcome of disease in the apparently immunocompetent is much more poorly understood. We describe a cohort of HIV uninfected Vietnamese patients with cryptococcal meningitis in whom underlying disease is uncommon, and relate presenting features of patients and the characteristics of the infecting species to outcome.

**Methods:**

A prospective descriptive study of HIV negative patients with cryptococcal meningitis based at the Hospital for Tropical Diseases, Ho Chi Minh City. All patients had comprehensive clinical assessment at baseline, were cared for by a dedicated study team, and were followed up for 2 years. Clinical presentation was compared by infecting isolate and outcome.

**Results:**

57 patients were studied. *Cryptococcus neoformans var grubii *molecular type VN1 caused 70% of infections; *C. gattii *accounted for the rest. Most patients did not have underlying disease (81%), and the rate of underlying disease did not differ by infecting species. 11 patients died while in-patients (19.3%). Independent predictors of death were age ≥ 60 years and a history of convulsions (odds ratios and 95% confidence intervals 8.7 (1 - 76), and 16.1 (1.6 - 161) respectively). Residual visual impairment was common, affecting 25 of 46 survivors (54.3%). Infecting species did not influence clinical phenotype or outcome. The minimum inhibitory concentrations of flucytosine and amphotericin B were significantly higher for *C. neoformans var grubii *compared with *C. gattii *(p < 0.001 and p = 0.01 respectively).

**Conclusion:**

In HIV uninfected individuals in Vietnam, cryptococcal meningitis occurs predominantly in people with no clear predisposing factor and is most commonly due to *C. neoformans var grubii*. The rates of mortality and visual loss are high and independent of infecting species. There are detectable differences in susceptibility to commonly used antifungal drugs between species, but the clinical significance of this is not clear.

## Background

*Cryptococcus neoformans *is the most important cause of invasive fungal disease worldwide. In the absence of HIV, neurological disease occurs most commonly in patients with some other cause of immunosuppression, such as malignancy or organ transplantation. Disease in these patients, and in HIV patients, is most usually due to infection with either *Cryptococcus neoformans var grubii *(*C. grubii*), or *Cryptococcus neoformans var neoformans *(*C. neoformans*) [1]. In the tropics and subtropics, *Cryptococcus gattii *(*C. gattii *)is a recognized cause of meningoencephalitis in the immunocompetent, and it is an emerging cause of disease in humans and animals in British Columbia [2-7].

Cryptococcal meningitis is rare in immunocompetent patients - treatment guidelines for these patients are based largely upon evidence from trials in HIV patients, although data suggest there are significant differences in clinical presentation and prognosis between the two groups[8-20]. We carried out a prospective descriptive study to determine the clinical presentation and outcome in HIV negative patients presenting to our hospital with cryptococcal meningitis.

## Methods

### Study design

Prospective observational descriptive study.

### Setting and participants

The study was conducted at the Hospital for Tropical Diseases, Ho Chi Minh City, the tertiary referral centre for infectious diseases in tropical southern Vietnam. The hospital serves a population of 40 million people and the well defined patterns of inter-hospital referral in Vietnam mean all patients in the catchment area with cryptococcal meningitis are likely be referred for treatment. All patients received initial treatment and follow-up on the dedicated central nervous systems (CNS) infection ward. Ethical approval for the study was from the Hospital for Tropical Diseases, Viet Nam and the Oxford University Tropical Ethics Committee, UK.

### Entry Criteria

The study was open to adult patients (age≥15 years) in whom HIV infection was excluded through 2 negative HIV antibody tests. Cryptococcal meningitis was defined as follows:

Either: Positive cerebrospinal fluid (CSF) India ink examination or positive CSF cryptococcal antigen test or positive CSF cryptococcal culture.

Or: a syndrome consistent with meningoencephalitis (1 or more of headache, neck stiffness, confusion, coma, convulsions, focal neurology) and culture or antigen positive blood.

Evidence of other central nervous system infection was an exclusion criterion.

Patients were recruited between November 1998 and July 2007. Written informed consent was obtained prior to study entry.

### Patient assessment

All patients were assessed daily by the dedicated study team. CSF was examined using standard methods including Gram's, Ziehl-Neelsen and India ink stains, and culture on blood, Sabouraud's and Lowenstein-Jensen media. HIV infection was excluded using Rapid Test and 2 HIV antibody tests (Determine HIV1/2, Abbott, Maidenhead, UK).

### Microbiology

*C. neoformans *was identified by the demonstration of budding encapsulated yeasts on India ink stain of cerebrospinal fluid (CSF). CSF pellets were cultured at 35°C on chocolate and blood agar, brain-heart infusion broth, and at 30°C and 35°C on Sabouraud's agar (Oxoid, Basingstoke, UK). Identification was confirmed through demonstration of the growth of characteristic colonies on bird seed agar and API 32C sugar assimilation tests (BioMerieux SA, Marcy l'Etoile, France). Isolates were stored at -30°C on Microbank plastic beads (Pro-Lab Diagnostics, Neston, Cheshire, UK) and later revived for speciation. *C. gattii *was identified by biotyping with Canavanine Glycine Bromothymol Blue agar, and isolates were further divided into eight molecular groups using Polymerase Chain Reaction- Restriction Fragment Length Polymorphism (PCR-RFLP) analysis of the *URA5 *gene[21]. Controls were provided by Dr Wieland Meyer, Westmead Millennium Institute for Medical Research, Sydney, Australia.

Semi-quantitative *Cryptococcus *antigen testing was performed using the Murex Cryptococcus Test (Remel, Lenexa, USA) according to the manufacturer's instructions. Sensitivity testing was performed using YeastOne Sensititre plates (Trek Diagnostic Systems, East Grinstead, UK) according to the manufacturer's instructions on the yeast isolated at presentation, prior to treatment. All plates were examined at 48 and 72 hours after incubation at 30°C.

### Treatment

Patients were treated according to Vietnamese national guidelines with intravenous amphotericin B 0.7 to 1mg/kg/day (Cipla, Mumbai, India) with oral flucytosine 100mg/kg/day (Valeant Pharmaceuticals, Versailles, France) at the physician's discretion, followed by fluconazole 400mg/day (Ranbaxy, Gurgaon, India). Treatment duration was at the discretion of the attending physician, guided by the clinical response of the patient. Raised intracranial pressure was managed by repeated lumbar puncture.

### Follow-up

Patients were followed for 2 years after hospital discharge.

### Statistical analysis

All data were entered into an electronic spreadsheet (Excel, Microsoft, Seattle, USA). Statistical analysis was performed using STATA version 9 (Statacorp, Texas, USA). For comparison of proportions of categorical or dichotomized parameters the chi-squared or Fisher's exact test were used. For continuous parameters the Mann-Whitney U-test was used. Forward step-wise logistic regression was performed to determine significant variables independently associated with outcome.

## Results

### Clinical Findings

Fifty-seven patients were recruited. Forty patients were transferred from other hospitals, and the median duration in these hospitals before transfer was 5 days (interquartile range 1 - 12 days). All had clinical syndromes consistent with cryptococcal meningitis and all underwent lumbar puncture. All had positive CSF India ink examination and positive CSF culture. History and examination findings on presentation are shown in Additional file 1: table S1. The duration of symptoms was unclear in one patient, due to a history of chronic headache (3 years) prior to diagnosis. The most frequent presenting symptoms were headache, fever and neck stiffness. Oculomotor nerve palsies and visual disturbance (decreased acuity or diplopia) were common on admission, affecting 18 (31.6%) and 27 (47%) patients respectively. Papilloedema was common, present in 22 of 55 patients (40%, data missing in 2 patients). Eleven patients (19%) had potentially immunosuppressive pre-existing health problems. Seven had taken more than three months of corticosteroid (nephrotic syndrome (2), Evan's syndrome (2), chronic arthritis (2) and systemic lupus erythematosus (1)). Two patients had cirrhosis, one had cirrhosis and renal impairment and one had diabetes.

Clinical investigations results are shown in Additional file 1: table S1. The baseline CSF opening pressure was frequently raised, greater than 30cmCSF in 33 of 54 patients (61%). Generally, CSF showed a predominance of lymphocytes with low blood glucose to CSF ratio. The CSF lactate was usually raised. CD4 counts were measured on a single occasion in 20 patients. 9 patients had CD4 counts of less than 400 cells/uL, of whom 3 were receiving treatment with corticosteroids, 2 had renal impairment and one had lupus.

### Imaging Results

56 of 57 patients had chest X-rays on admission. 12 were abnormal - six showed infiltrative shadowing, five were consistent with old resolved pulmonary TB, and one showed bilateral pleural effusions. 39 scans were performed on 36 patients (26 CT brain scans (Toshiba XVIsion, Toshiba, Japan), 13 Magnetic Resonance (General Electric, UK, 1.5T), (MR) brain scans. Abnormalities included ventricular dilatation, infarction, focal abnormalities consistent with cryptococcomas and meningeal enhancement.

### Microbiological Findings

**Figure 1:**
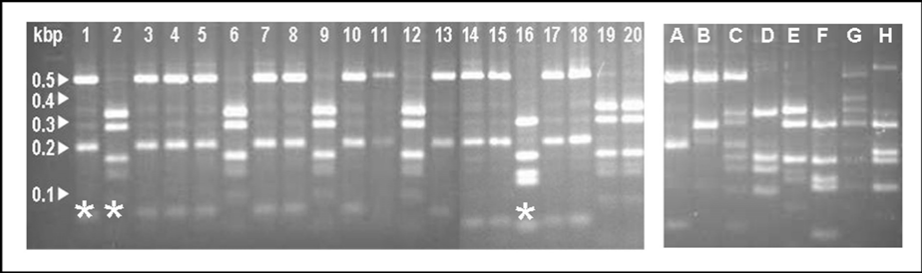
**Representative PCR-RFLP profiles of the *URA5 *gene of 20 *Cryptococcus *isolates and 8 control strains A - H**. Footnote: A = VN1, B = VN2, C = VN3, D = VN4, E = VG1, F = VG2, G = VG3, H = VG4 * denotes examples of detected molecular groups.

All patients had characteristic encapsulated yeasts seen on cerebrospinal fluid India ink examination, and positive CSF culture. 52 patients had CSF cryptococcal antigen testing - all were positive (median titre 1/256, range 1/4 to 1/32768). Ten of 56 patients (18%) had blood cultured and were fungaemic, and 44 of 53 patients tested had cryptococcal antigen detectable in blood, giving this simple test a sensitivity of 83%. 35 isolates were revivable for PCR-RFLP typing and antifungal sensitivity testing. 10 were *C. gattii*, and 25 were *C. neoformans *var *grubii*. All the *C. neoformans *var *grubii *fell into molecular group VN1. One *C. gattii *isolate was molecular group VG2, the rest were VG1 (figure 1). One of 10 (10%) patients with *C. gattii *infection had underlying disease, versus 7 of 25 (28%) patients with *C. neoformans *var *grubii *infection (p = 0.45, Fisher's exact test).

### Susceptibility Testing

Antifungal susceptibility results (Table 1) were interpretable for 34 of the 35 isolates (1 isolate grew too slowly despite incubation at a range of temperatures). Breakpoints for *Cryptococcus neoformans *are not defined; we present the minimum inhibitory concentrations (MIC) of drug that inhibited 90% (MIC90) and geometric mean MICs (GMICs) of the isolates according to species (Table 1). The GMICs of flucytosine and amphotericin were significantly higher for *C. neoformans *var *grubii *isolates than for *C. gattii *(8.23 vs 1.85 μg/ml, (95% CI 7.22 - 9.37 and 1.18 - 2.90, p < 0.001)) and 0.714 μg/ml vs 0.451 μg/ml, (95% CI 0.617 - 0.826 and 0.341 - 0.598 p = 0.01) respectively. There were no differences in the MICs of any other azole by species. We could not detect any association between antifungal susceptibility and clinical outcome.

**Table 1:** Features of 35 cryptococcal meningitis cases according to infecting species.

		*C. neoformans *var *grubii*	*C. neoformans *var *gattii*		
		(n = 25)	(n = 10)		
**Variable**		**Value**	**Value**	**Odds ratio**	**P Value**
		**(Range or %)**	**(range or %)**	**(95% CI)**	

Median Age (range)		31 (15 - 74)	41 (18 - 53)		0.47
Male Sex		10 (40)	5 (50)	1.48 (0.26 - 8.4)	0.71
Underlying Disease		7 (28)	1 (10)	0.29 (0.01 - 2.9)	0.39
**Symptoms**					
Fever		20 (80)	9 (90)	2.2 (0.2 - 117)	0.64
Headache		19 (90)	8 (89)	0.85 (0.04 - 55.8)	1.0
Vomiting		9 (36)	5 (50)	1.75 (0.31 - 10.1)	0.47
Blurred vision		12 (52)	4 (40)	0.62 (0.10 - 3.47)	0.71
Diplopia		4 (20)	2 (20)	1.0 (0.95 - 10.9)	1.0
Convulsions		4 (16)	1 (10)	0.59 (0.01 - 7.2)	0.59
**Signs**					
GCS < 11/15		7 (28)	1 (10)	0.29 (0.01 - 2.9)	0.39
Neck Stiffness		22 (88)	8 (80)	0.56 (0.05 - 7.8)	0.61
Focal Neurological Signs		7 (28)	5 (50)	2.5 (0.4 - 15.0)	0.26
Papilloedema		5 (21)	5 (50)	3.63 (0.6 - 24.3)	0.11
**Investigations**					
Abnormal Chest X-ray		8 (32)	2 (20)	0.54 (0.05 - 3.7)	0.69
Abnormal Brain Imaging^a^		7 (58)	7(100)	-	0.13
Haematocrit		37.8 (25.3 - 51.4)	38.8 (31.1 - 46)		0.65
Blood White Cell Count (×10^9/ml)		11.5(3.3 - 26.6)	13.6 (7.1 - 26.1)		0.37
Platelet count (×10^9/L)		256 (56.9 - 356)	305 (140 - 487)		
CSF opening pressure (cmCSF)^b^		34.2 (2 - > 40)	30.5 (15 - > 40)		0.44
CSF White Cell Count/ml		287 (2 - 1080)	279 (12 - 480)		0.18
CSF Lymphocyte %^c^		53 (12 - 97)	56 (19 - 93)		0.74
CSF Protein (g/L)		1.28 (0.35 - 2.63)	1.25 (0.4 - 1.94)		0.9
Log2 CSF Cryptococcal antigen titre^5^		7.4 (3 - 15)	8.6 (2 - 14)		0.5
Lactate (mmol/L)		6.2 (1.6 - 22.5)	4.2 (2 - 8.4)		0.052
**Antifungal MICs (mg/L)**					
Amphotericin	GM(95%CI)	0.71 (0.62 - 0.83)	0.45 (0.34 - 0.60)		0.01
	MIC90	1.0	0.5		
Flucytosine	GM(95%CI)	8.23 (7.22 - 9.37)	1.85 (1.18 - 2.90)		< 0.001
	MIC90	16/0	4.0		
Fluconazole	GM(95%CI)	6.96 (5.11 - 9.49)	4.54 (2.44 - 8.42)		0.19
	MIC90	16.0	16.0		
Itraconazole	GM(95%CI)	0.11 (0.08 - 0.14)	0.9 (0.05 - 0.14)		0.48
	MIC90	0.26	0.26		
Voriconazole	GM(95%CI)	0.07 (0.05 - 0.09)	0.06 (0.04 - 0.51)		0.85
	MIC90	0.13	0.26		
Posaconazole	GM(95%CI)	0.13 (0.10 - 0.17)	0.10 (0.06 - 0.17)		0.336
	MIC90	0.26	0.26		

^a^N = 26, ^b^N = 33, ^c^N = 32, ^d^N = 31

GM = Geometric Mean

MIC90 = minimum inhibitory concentration 90

### Outcome

11/57 (19%) patients died during admission, and the median time to death was 14 days (4 -156 days). Three patients withdrew from the study (one after 12 and two after 37 days of treatment). The median duration of treatment was 131 days (range: 51- 426 days). One (10%) of 10 patients with *C. gattii *died compared with 6 (24%) of 25 patients with *C. neoformans *var *grubii *infection (p = 0.65). Complications at discharge included four patients with bilateral blindness (no light perception), 4 with uni-ocular blindness and one with complete visual and hearing loss. All cases of blindness occurred in patients with opening CSF pressures of ≥ 30cm CSF - 9 of 28 patients with opening pressure ≥ 30cm CSF developed blindness compared with none of 17 patients with opening pressure < 30 cm CSF, p = 0.008). Five of 9 patients with blindness (56%) had papilloedema at baseline compared with 14 (39%) of 36 patients with no visual loss (odds ratio 1.85, 95% confidence interval 0.33 - 11.09, p = 0.47). There were no differences in the rates of papilloedema, blindness or opening CSF pressure > 30cmCSF by infecting species. Fourteen patients had other neurological deficits including blurred vision and/or cranial nerve palsies.

On univariate analysis the admission findings associated with death were presence of underlying disease, history of convulsions, Glasgow Coma Score ≤ 11, age ≥ 60 years, lower platelet count, CSF cryptococcal antigen titre ≥ 1/512 and CSF yeast cell count > 400 cells/ml. Six of 21 (29%) patients with CSF opening pressure < 30cm CSF died, compared with 7 of 33 (21%) patients with opening pressures ≥ 30cm CSF (odds ratio 0.68, 95% confidence interval 0.16 - 2.94, p = 0.745). Two of 9 patients with CD4 lymphopenia died.

Multivariate analysis was limited by the small number of deaths. We tested the interaction between easily measured clinical parameters (age ≥ 60 years, underlying disease, Glasgow coma score < 11 and convulsions) with outcome. Only age ≥ 60 years and a history of convulsions were independently associated with death (Additional file 1: table S1). Length of history had no impact on outcome. We could detect no differences in clinical phenotype or outcome according to infecting species.

Two years after diagnosis a further 4 patients had died (total deaths 15 of 57, 26.3%). 14 patients (24.6%) had made complete recoveries with no residual deficit. One died due to multi-drug resistant tuberculosis, complicated by relapse of cryptococcal disease. Three patients with underlying disease continued on long term fluconazole prophylaxis. It was not possible to determine the 2 year outcome in 9 of the 57 patients.

## Discussion

Cryptococcal meningitis in HIV uninfected patients in tropical Viet Nam is most commonly due to *Cryptococcus neoformans *var *grubii*. Most patients (81%) have no concomitant immunosuppressive disease, although CD4 lymphopenia was detected in 9 patients, 3 of whom had no other underlying conditions.

*C. neoformans *var *grubii*, the commonest cause of cryptococcal meningitis since the HIV epidemic, usually occurs in patients with an underlying immunosuppressive condition and has rarely been reported in the absence of underlying disease, in southeast Asia or elsewhere [2,12,14,22-27]. Classical teaching would suggest that, in our tropical location, more cases would have been due to infection with *Cryptococcus gattii*[28,29]. Against expectation, in our series *C. neoformans *var *grubii *infection outweighed *C. gattii *infection 2.5-fold. We could only revive isolates for speciation from 35 patients, but since the study has finished we continue to isolate *C. neoformans *var *grubii *and *C. gattii *from HIV uninfected patients in the ratio 3:1. We could not detect differences in the rate of underlying disease according to infecting species, although our study lacked power since there were only 10 cases of *C. gattii *infection.

All patients in our study had positive CSF India ink examination and culture - a rate higher than generally reported in the literature [1]. This may be a reflection of the large volumes of CSF (5 - 10 mls) routinely taken from patients with suspected meningitis in our hospital in order to exclude tuberculous meningitis.

In the tropical Northern Territories of Australia the incidences of *C. gattii *and *C. neoformans *infection are equal[30]. In Queensland the experience is more like our own - in a 3 year period *C. neoformans *infections outweighed *C. gattii *5 fold, although the total number of cases is not clear from the report[30]. However, it is not clear whether these cases were meningoencephalitis or extra-neural disease, and the varietal form of *C. neoformans *was not described for the majority of isolates.

All the *C. neoformans *var *grubii *strains from our series were of *URA5-*RFLP molecular type VN1, as also recently reported from China [31]. In that series, there was remarkable genetic homogeneity between the VN1 isolates, perhaps representing a strain in Asia with increased ability to cause infection in the immunocompetent.

The significance of CD4 lymphopenia in our patients is uncertain. Only 20 patients had the investigation, and the test was not performed serially. Thus it is not clear whether it represents a transient phenomenon as a consequence of infection, or whether it represents a genuine pre-existing immune deficit, such as idiopathic CD4 lymphopenia, known to be associated with an increased risk of cryptococcosis [32]. HIV infection is unlikely since each patient received at least 2 HIV antibody tests.

While cryptococcal meningitis remains rare in HIV uninfected patients, when it occurs the consequences are severe [33]. The death rate in our patients is 19% at hospital discharge, and blindness affects 20% of survivors. Most patients were admitted to provincial or district hospitals before transfer to our centre, and delay in diagnosis may play a role in the high death rate seen in this disease, although we did not find length of history to be associated with death. The independent variables associated with death were age and convulsions. We did not find the presence of underlying disease to be independently associated with outcome, although analysis was limited by the low number of deaths. CSF opening pressure at baseline was highly associated with development of blindness, but there was no association with papilloedema, which probably reflects the fact that papilloedema is a late indicator of raised intracranial pressure. However, it could be a function of the size of our study. Only 25% of patients made a complete recovery.

Historically, the disease phenotypes due to infection with *C. neoformans *var *grubii *and *C. gattii *are considered to be different. We found no difference in clinical phenotype between the 2 infections, and the pattern of disease seen in our cases mimics that seen in case series of *C. gattii *infection published from Papua New Guinea[17,27]. Like us, Seaton *et al *found presence of convulsions to be a predictor of death in patients with *C. gattii *meningitis. In addition, the rate of blindness in our patients is similar to that reported for *C. gattii *infection in immunocompetent adults, and higher than in HIV infected patients. The lack of difference in the clinical phenotypes of disease according to infecting species and the difference in rates of visual loss between HIV positive and negative patients support the hypothesis that host immune status is key in determining clinical phenotype in cryptococcal disease[30]. There are retrospective data suggesting that immune mediating drugs (corticosteroids) may reduce the risk of blindness in *C. gattii *infection [34,35]. The similar clinical phenotype seen in our predominantly *C. neoformans *var *grubii *infected patients suggests that this may be a worthwhile hypothesis to test in our patients.

There are no established breakpoints for anti-fungal drugs and *C. neoformans*, which makes the interpretation of anti-fungal MICs difficult [36-39]. The geometric mean MICs of flucytosine and amphotericin B were statistically significantly higher for *C. neoformans *var *grubii*. This is consistent with the experience in Malaysia, but differs from reports from Brazil and Taiwan which found *C. gattii *to be inherently less susceptible to amphotericin B and flucytosine [39-41]. Thompson found no difference in MICs by species, but was comparing strains from multiple geographic regions[42]. Our data are reassuring, suggesting that it is safe to extrapolate treatment doses for our patients with *C. gattii *from trials in *C. neoformans *infection. However, the clinical significance of differences in MICs of flucytosine and amphotericin B remain unclear. MIC data were available for only 7 of the isolates from the 11 patients that died - we could not demonstrate any correlation between the MIC of any drug and patient outcome.

Recently, *Cryptococcus gattii *has been seen to establish new ecological niches, with subsequent successful human-mediated dispersal [43]. It is possible that disease incidence in the immunocompetent will increase [44,45]. There has been little change in the treatment of cryptococcal meningitis over the past 10 years. Amphotericin B remains a key component of treatment, but therapy is protracted, expensive and difficult to administer. Combined with flucytosine, it has been associated with more rapid CSF sterilisation in HIV patients, and more recently slower rates of CSF sterilisation have been shown to be associated with an increased risk of death [46,47]. The identification of a reliable surrogate marker of outcome in cryptococcal meningitis enables anti-fungal therapeutic trials to be undertaken with smaller numbers of patients, and it should now be possible to answer clinical questions in groups where disease is rare, such as HIV uninfected patients. However, differences in complication rates, such as blindness, between HIV infected and uninfected patients may be due to differences in disease pathogenesis (perhaps immune mediated) and clinical endpoint trials, although difficult, may still be necessary.

## Conclusions

In HIV uninfected individuals in Vietnam, cryptococcal meningitis occurs predominantly in people with no clear predisposing factor and is most commonly due to *C. neoformans var grubii*. The pattern of disease is similar to that described in patients with *C. gattii *meningitis, suggesting underlying immune status is key in generating clinical phenotype. The rates of mortality and visual loss are high and independent of infecting species.

## Competing interests

The authors declare that they have no competing interests.

## Authors' contributions

JJF, TTHC, NHM, NHP, HDN, LVC, DXS, TTH, DGL & JND conceived the study, participated in its design, coordination, collected data, and helped to draft the manuscript. TTHC analysed the data. VAD, PTD, JIC, and JND performed the classical mycology and MIC testing. VAD, SB and JND performed the molecular typing. JND wrote the final manuscript. All authors read and approved the final manuscript.

## Funding

The study was funded by the Wellcome Trust (UK) and JND was supported by a British Infection Society Fellowship. The funding sources had no involvement in study design, collection, analysis or interpretation of the data, writing of the report, or the decision to submit for publication.

## Pre-publication history

The pre-publication history for this paper can be accessed here:

http://www.biomedcentral.com/1471-2334/10/199/prepub

## Supplementary Material

Additional file 1 **Table S1.** Presenting features of 57 consecutive cryptococcal meningitis patients by outcome. Click here for file
