# Supplementary material for: A prospective descriptive study of cryptococcal meningitis in HIV uninfected patients in Vietnam - high prevalence of Cryptococcus neoformans var grubii in the absence of underlying disease
Source: BMC Infect Dis. 2010 Jul 9;10:199. doi: 10.1186/1471-2334-10-199 (PMC2910700; doi:10.1186/1471-2334-10-199)
Supplement: Additional file 1 — Table S1. Presenting features of 57 consecutive cryptococcal meningitis patients by outcome. [file 1471-2334-10-199-S1.DOC]

| **Table S1. Presenting features of 57 consecutive cryptococcal meningitis patients by outcome.** | | | | | | |
| --- | --- | --- | --- | --- | --- | --- |
|  | **Survivors**  **N = 46** | **Died**  **N = 11** | **Univariate Analysis** | | **Multivariate Analysis** | |
| **Variable** | **Value**  **(Range or %)** | | **Odds ratio**  **(95% CI)** | **P Value**  **(χ^2^ unless indicated)** | **Odds ratio (95% CI)** | **P Value** |
| Age years | 30  (15 – 75) | 53  (21 – 75) |  | **0.005** |  |  |
| Age ≥ 60 years | 4  (8.7) | 5  (45.6) | **8.8**  **(1.8 – 42.0)** | **0.008** | **8.7**  **(1 – 76)** | **0.049** |
| Male Sex | 25  (54.4) | 6  (54.6) | 1.0  (0.22 – 4.8) | 0.99 |  |  |
| Underlying Disease | 5  (10.9) | 6  (54.4) | **9.3**  **(1.70 – 57.1)** | **0.004** | 4.0  (0.5-29.7) | 0.18 |
| **Symptoms** |  |  |  |  |  |  |
| Fever | 35 (76.1) | 9 (81.8) | 1.4  (0.23 – 15.3) | 0.68 |  |  |
| Headache | 46 (100) | 11 (100) |  |  |  |  |
| Vomiting^a^ | 37 (82.2) | 5 (45.5) | **0.2**  **(0.04 – 0.07)** | **0.02** |  |  |
| Blurred vision^b^ | 21 (50) | 1 (14.3) | 0.2  (0.00 – 1.6) | 0.11 |  |  |
| Diplopia^c^ | 11 (25) | 0 | 0 (0 – 1.57) | 0.17 |  |  |
| **Signs** |  |  |  |  |  |  |
| GCS < 11/15 | 5 (10.9) | 6 (54.6) | **9.8**  **(2.1 – 44.4)** | **0.003** | 7.5  (0.95–9.0) | 0.056 |
| Neck Stiffness | 39 (84.8) | 10 (90.9) | 1.8  (0.18 – 88.9) | 0.89 |  |  |
| Cranial nerve palsy |  |  |  |  |  |  |
| 3^rd^ nerve^d^ | 4 (8.7) | 2(18.8) | 2.3  (0.18 – 19.05) | 0.35 |  |  |
| 6^th^ nerve^d^ | 16 (34.8) | 1 (9.1) | 0.2  (0 – 1.57) | 0.09 |  |  |
| 7^th^ nerve^d^ | 4 (8.7) | 3 (27.7) | 3.9  (0.5 – 27.76) | 0.09 |  |  |
| Papilloedema^d^ | 19 (42.2) | 3 (30) | 0.6  (0.08 – 3.02) | 0.47 |  |  |
| Convulsions^d^ | 3 (6.52) | 4 (45.5) | **11.9**  **(2.3 – 63.3)** | **0.004** | **16.1**  **(1.6 – 161)** | **0.01** |
| Hemiparesis^d^ | 2 (4.35) | 1 (9.1) | 2.2  (0.03 – 45.5) | 0.52 |  |  |
| Paraparesis^d^ | 3 (4.35) | 0 (0) | 0  (0 – 8.47) | 0.48 |  |  |
| **Investigations** |  |  |  |  |  |  |
| Abnormal Brain Imaging | 24  (72.7) | 3  (60) | 0.56  (0.05 – 7.9) | 0.56 |  |  |
| Haematocrit | 45  (23.7 – 51.4) | 34.2  (30.9 – 46) |  | 0.87 |  |  |
| Blood WCC (X10^9^/L) | 10.6  (0.54 – 26.7) | 8.36  (3.33 – 26.6) |  | 0.72 |  |  |
| Neutrophils (%) | 82.3  (57.1 – 95) | 80  (65 – 89) |  | 0.33 |  |  |
| Lymphocytes (%) | 9.9  (3.6 – 32) | 12.4  (5.11 – 22) |  | 0.32 |  |  |
| CD4 count (X10^6^/L) | 417  (43 – 1002) | 222  (34 – 757) |  | 0.71 |  |  |
| CD8 count (X10^6^/L) | 289  (22 – 538) | 244  (69 – 278) |  | 0.46 |  |  |
| Platelets (X10^9^/L) | 292.5  (39.7 – 501) | 180  (35 – 380) |  | **0.007** |  |  |
| Fungaemia^d^ | 8 (17.4) | 2 (20%) | 1.2 (0.1 – 7.8) | 0.85 |  |  |
| Positive CrAg^e^ | 35  (81.4) | 9  (90) | 2.1 (0.21 – 101) | 0.51 |  |  |
| CSF Opening Pressure (cmCSF) | 30.1  (26.3 – 33.9) | 28.8  (19.8 – 37.8) |  | 0.62 |  |  |
| CSF WCC (X10^9^/L) | 143  (1 – 105) | 160  (7 – 1080) |  | 0.91 |  |  |
| Lymphocytes % | 67  (10 – 100) | 62.5  (12 – 85) |  | 0.21 |  |  |
| Protein (g/dL) | 1.12  (0.35 – 3.5) | 1.5  (0.41 – 2.88) |  | 0.24 |  |  |
| Lactate mmol/L | 4.4  (1.6 – 10) | 5.1  (3.2 – 22.5) |  | 0.11 |  |  |
| CSF:blood glucose <0.4 | 35  (78.3) | 10  (90.9) | 2.77  (0.31 – 132.4) | 0.34 |  |  |
| CrAg Titre ≥ 1/512^f^ | 13  (30.95) | 7  (70) | 5.2  (1.15 – 23.8) | **0.031** |  |  |
| CSF yeast cells> 400/ml^g^ | 3  (14.3) | 7  (70) | 8  (1.15 – 55.25) | **0.035** |  |  |
| **Species^h^** |  |  |  |  |  |  |
| *Var grubii* | 20  (76.9) | 6  (23.1) | 0.34  (0.01 – 3.47) | 0.65 |  |  |
| *Var gattii* | 10  (90.9) | 1  (9.1) |  |  |  |  |
| ^a^N=56 ^, b^N = 49, Fisher’s Test, ^c^N = 52, Fisher’s Test, ^d^ = Fisher’s Test, ^e^N = 53, ^f^N = 52, ^g^N = 28, Fisher’s Test, ^h^ = Fisher’s Test, CrAg = Cryptococcal Antigen, WCC = White Cell Count | | | | | | |
